# Alpha-ketoglutarate supplementation improves hyperglycemia and attenuates the decrease in GLUT4 and PGC-1α proteins in adipose tissue of streptozotocin-high-fat diet-induced diabetic mice

**DOI:** 10.1017/jns.2025.10059

**Published:** 2025-12-17

**Authors:** Ai Takemura, Yutaka Matsunaga, Shota Hajime, Wenxin Wang, Yumiko Takahashi, Hideo Hatta

**Affiliations:** 1 Department of Sports Sciences, The University of Tokyo, Tokyo, Japan; 2 Ritsumeikan Global Innovation Research Organization, Ritsumeikan University, Shiga, Japan; 3 Department of Sports Medicine and Science, Kurume University, Fukuoka, Japan

**Keywords:** Adipose tissues, Alpha-ketoglutarate, Diabetes, Skeletal muscles, AKG, Alpha-ketoglutarate, GLUT4, glucose transporter 4, T2DM, type 2 diabetes mellitus, BCAA, branched-chain amino acid, HFD, high-fat diet, ICR, Institute of Cancer Research, CON, control, DM, diabetes, STZ, streptozotocin, TA, tibialis anterior, BAT, brown adipose tissue, ELISA, enzyme-linked immunosorbent assay, HOMA-IR, homeostatic model assessment of insulin resistance, PGC-1α, peroxisome proliferator-activated receptor γ coactivator 1 alpha, UCP1, uncoupling protein 1, PRDM16, PRD1-BF-1-RIZ1 homologous domain containing protein 16, OXPHOS, oxidative phosphorylation, NDUFB8, NADH dehydrogenase (ubiquinone) 1β subcomplex 8, SDHB, succinate dehydrogenase complex subunit B, UQCRC2, ubiquinol-cytochrome c reductase core protein II, ATP5A, ATP synthase, H+ transporting, mitochondrial F1 complex, α-subunit, BCAT2, BCAA transaminases 2, SD, standard deviation, AMPK, AMP-activated protein kinase, mTORC1, mechanistic target of rapamycin complex 1, BCKAs, branched-chain keto acids

## Abstract

Alpha-ketoglutarate (AKG) is a well-known intermediate of the tricarboxylic acid cycle and plays an important role in the catabolism of branched-chain amino acids (BCAAs: leucine, isoleucine, and valine). While previous study suggested that AKG enhances glucose metabolism, its effect on the adaptation of muscles and adipocytes has not been well studied in diabetic condition. This study aimed to determine whether AKG improves glucose metabolism in the skeletal muscles and adipose tissues in diabetic mice. Male institute of cancer research mice were divided into control, diabetic, and diabetic + AKG groups. Diabetes (DM) was induced by a high fat diet consumption and streptozotocin (STZ) injection. Mice in the DM + AKG group were administered 1% AKG in drinking water for 6 weeks. The non-fasting plasma glucose level was significantly higher in the diabetic group than that in the control and DM + AKG groups (*P* < 0.05). No significant difference was observed in glucose transporter 4 (GLUT4) protein levels in the muscles between the DM and DM + AKG groups. AKG supplementation attenuated the decrease in peroxisome proliferator-activated receptor γ coactivator 1 alpha and GLUT4 protein levels in inguinal and epididymal adipose tissues in diabetic condition. In conclusion, the study findings suggested that AKG supplementation increased protein levels related to mitochondrial biogenesis and glucose transporters in adipocyte tissue accompanied with improved whole-body glucose metabolism in STZ and high-fat diet-induced diabetic mice.

## Introduction

High caloric intake and low physical activity lead to excessive triglyceride accumulation in adipocytes,^(1)^ inducing obesity and/or type 2 diabetes mellitus (T2DM).^(2)^ While skeletal muscles are known as major insulin target tissues and play an important role in glucose regulation throughout the body, responsible tissues for glucose disposal stimulated via insulin are not only the skeletal muscles, but also the adipose tissues.^(3)^ The rate-controlling step for insulin-stimulated glucose utilization in the skeletal muscle and adipose tissues is glucose transport mediated by GLUT4.^(4,5)^ GLUT4 plays an essential role in glucose uptake in skeletal muscle and adipose tissue, and its translocation to the cell membrane is regulated by insulin.^(4,5)^ In the diabetes (DM) and obesity, GLUT4 level decreased in the skeletal muscle and adipose tissues.^(6–8)^ Hyperglycemia and the decreased GLUT4 level in the skeletal muscle in diabetic condition were improved by exercise training.^(8)^ Improvement of the glucose disposal via GLUT4 in the skeletal muscles and adipose tissues improve the glucose metabolism in whole-body level. In addition, glucose is utilized in adipose tissue for energy production and fatty acid biogenesis that requires mitochondrial metabolism.^(9)^ PGC-1α is a master regulator of mitochondrial biogenesis,^(10)^ while PRDM16 controls adipose tissue development and, together with PGC-1α, induces UCP1 expression.^(11,12)^ UCP1 uncouples mitochondrial respiration to promote thermogenesis and contributes to systemic glucose homeostasis.^(13)^

Recently, some previous studies reported that elevated plasma branched-chain amino acid (BCAAs: leucine, isoleucine, and valine) concentrations are related to insulin resistance, resulting in a high diabetic risk.^(14,15)^ Leucine induced the suppressive effect on insulin-stimulated glucose transport in the skeletal muscle-derived L6 myotubes.^(16)^ Moreover, high-fat diet (HFD) with BCAA intake aggravated blood glucose control compared with HFD intake without BCAA in human.^(17)^ Alpha-ketoglutarate (AKG, IUPAC name: 2-oxopentanedioic acid), an intermediate of the tricarboxylic acid cycle, is known a substrate for BCAA transaminases and plays an important role in BCAA catabolism.^(18)^ Previous study indicated that lower intracellular AKG concentration and higher BCAA concentration were observed in type 2 DM and obesity compared with healthy group.^(14,19)^ We hypothesized that supplementation of AKG may improve DM via enhancing BCAA catabolism.

Moreover, previous study showed that dietary AKG induced an adaptation in the adipose tissue.^(20)^ Dietary AKG promotes beige adipogenesis in middle-aged mice.^(20)^ Beige adipocytes contain higher amount of mitochondria than white adipocytes and take up glucose and fatty acids to increase heat production in mitochondria.^(21)^ In white adipose tissues, it has been reported that exercise and some types of nutrients increased expression of genes responsible for regulation of glucose uptake and mitochondrial biogenesis and caused a beiging of adipocyte.^(22,23)^ These metabolic changes in the adipose tissue play a key role in the regulation of glucose metabolism throughout the body.^(24)^

Although previous studies indicated that AKG enhances glucose metabolism in DM and obese rodents,^(25,26)^ its effect on the adaptations in muscles and adipocyte tissues has not been well studied. For example, dietary AKG supplementation has been reported to lower plasma glucose, improve glucose tolerance, and reduce white adipose tissue mass in HFD-induced obese rats.^(26)^ These findings indicate that AKG can ameliorate metabolic disturbances associated with obesity, although its tissue-specific effects remain unclear. We hypothesized that AKG supplementation improves control of blood glucose by (1) degradation of BCAA concentration in the blood and (2) altering protein levels related with glucose metabolism of skeletal muscle and white adipose tissue.

## Methods

### Animals

Male Institute of Cancer Research (ICR) mice (8-week-old; Japan SLC, Inc., Tokyo, Japan) were used in this study. Mice were housed individually in standard cages under a 12:12 h light/dark cycle (dark period: from 07.00 to 19.00). This study was conducted at 22°C as the housing temperature. Some studies have argued that housing temperature of mice is approximately 30°C and that the current practice of housing mice at 20–22°C impairs the suitability of mice.^(27,28)^ However, a comparison of the thermoregulatory curves of humans and mice, combined with the temperatures routinely selected by humans, suggests that the optimal temperature is in the range of 23–25°C for individually housed mice with bedding and nesting material.^(29)^ All experiments were approved by the Animal Experimental Committee of the University of Tokyo (approval number: 2021-12).

### Experimental procedures

We randomly divided 8-week-old mice into normal control (CON, n = 7), diabetes (DM, n = 9), and diabetes and AKG supplementation (DM + AKG, n = 10) groups. In the DM and DM + AKG groups, mice were fed a high fat diet (HFD, 60% energy from fat, D12492, Research Diets, NJ, USA) from 9 to 27 weeks of age *ad libitum*, and freshly prepared streptozotocin (STZ; 40 mg/kg, Sigma, MO, USA) was intraperitoneally injected on three consecutive days at 11 weeks of age as a T2DM model based on previous study.^(30)^ In the CON group, mice were fed a control diet (10% energy from fat, D12450J, Research Diets, NJ, USA) from 9 to 27-weeks-old and water *ad libitum* and were simultaneously administered the same volume of saline as the STZ injection. Mice in the CON and DM groups were provided with water *ad libitum*. Mice in the DM + AKG groups were provided with water from 9 to 21 weeks of age and 1% AKG in drinking water from 21 to 27 weeks of age. Blood samples for measuring fasting glucose and insulin levels were collected via the tail veins of mice at the 26-week-old mice. Blood samples for measuring non-fasting glucose, AKG, and BCAA levels were collected from the inferior vena cava in the 27-week-old mice anesthetized by using isoflurane. The soleus, plantaris, tibialis anterior (TA) and gastrocnemius muscles and inguinal, visceral, retroperitoneal, epididymal and mesenteric fats and brown adipose tissue (BAT) were taken. The soleus and plantaris muscles and inguinal and epididymal fats rapidly frozen in liquid nitrogen and stored at –80°C until further analysis. The weight of visceral fats was calculated by adding retroperitoneal and epididymal, mesenteric fats.

### Analytical methods

#### Water consumption

Water consumption for 24 h in the three groups was measured at 26 weeks of age. Water consumption was calculated by determining the difference in the bottle weight between the beginning and end of the trial.

#### Blood analysis

The collected blood samples were centrifuged (4°C, 5000×g, 10 min), and the plasma fraction was rapidly frozen in liquid nitrogen and stored at –80°C until further analysis.

Plasma glucose concentrations were determined enzymatically using a Glucose CII Test kit (Cat# 439-90901, Wako, Tokyo, Japan). Plasma insulin concentrations were measured using an enzyme-linked immunosorbent assay (ELISA) kit (Morinaga Ultra-Sensitive Mouse/Rat Insulin ELISA Kit, MIoBS, Yokohama, Japan). The homeostatic model assessment of insulin resistance (HOMA-IR) was calculated using the following equation: HOMA-IR = fasting glucose level (mg/dL) × fasting insulin level (μU/mL)/405.^(31)^ Plasma AKG concentrations were measured using a colorimetric assay kit (Alpha Ketoglutarate Assay Kit; Abcam, Cambridge, UK). Plasma BCAA concentrations were measured using a colorimetric assay kit (Branched Chain Amino Acid Colorimetric Assay Kit; BioVision, MA, USA).

#### Western blotting

Frozen skeletal muscle and adipocyte tissues were homogenized in radioimmunoprecipitation assay lysis buffer (20–188, Millipore, MA, USA) containing a protease inhibitor (1183617001, Complete Mini EDTA-free, Roche Life Science, Indianapolis, IN, USA). After centrifugation at 1,500×*g* for 20 min at 4°C, the supernatant was collected, and the protein concentrations of the samples were determined using a BCA Protein Assay Kit (T9300A, Takara, Shiga, Japan). Protein samples (10 μg) and a pre-stained molecular weight marker (Bio-Rad, Hercules, CA, USA) were electrophoresed on 12% sodium dodecyl sulfate-polyacrylamide gels for 40 min at 200 V. Proteins were transferred to polyvinylidene difluoride membranes. The membranes were blocked with Blocking One (03953-95, Nacalai Tesque, Kyoto, Japan) for 5 min at room temperature. The membranes were incubated overnight at 4°C with the following primary antibodies: anti-glucose transporter 4 (GLUT4, 07–1404, Merck, Tokyo, Japan), anti-peroxisome proliferator-activated receptor γ coactivator 1 alpha (PGC-1α, 516557, Merck), anti-uncoupling protein 1 (UCP1, ab10983, Abcam), PRD1-BF-1-RIZ1 homologous domain containing protein 16 (PRDM16, ab303534), anti-oxidative phosphorylation [OXPHOS; NADH dehydrogenase (ubiquinone) 1β subcomplex 8 (NDUFB8), succinate dehydrogenase complex subunit B (SDHB), ubiquinol-cytochrome c reductase core protein II (UQCRC2), ATP synthase, H+ transporting, mitochondrial F1 complex, α-subunit (ATP5A), ab110413, Abcam], and anti-BCAA transaminases 2 (BCAT2, 16417-1-AP, Proteintech, IL, USA). Thereafter, membranes were incubated for 60 min at room temperature with the following secondary antibodies: a goat anti-rabbit IgG (H&L) (A102PT, American Qualex, CA, USA) and a goat anti-mouse IgG (H&L) (A106PU, American Qualex).

Proteins were detected using Pierce ECL Western blotting Substrate (Thermo Fisher Scientific, MA, USA) and visualized using ChemiDoc XRS (170–8071, Bio-Rad). The blots were quantified using Quantity One software (170–9600, Bio-Rad). Consistent loading was verified using Ponceau S solution (P7170; Sigma-Aldrich, MO, USA) as previously described.^(32,33)^

### Statistical analyses

All mice were included in the analysis. Grubbs’ test was used to detect outliers, which were discarded for subsequent analyses. Data normality and homoscedasticity were confirmed using the Shapiro-Wilk test and Bartlett’s test, respectively. Data are expressed as the mean ± standard deviation (SD). Differences among the CON, DM, and DM + AKG groups were evaluated using one-way analysis of variance followed by Tukey’s multiple comparison test. *P* < 0.05 was considered to be statistically significant. Statistical analyses were performed using GraphPad Prism software (ver. 9.0, Macintosh, GraphPad Software, La Jolla, CA).

## Results

### Food and water consumption, body weight, and tissue weight

No difference in average calorie intake per week relative to body weight was observed between three groups (CON, 3.3 ± 0.5; DM, 2.8 ± 0.2; DM + AKG, 3.1 ± 0.7 kcal/week/body weight), from 9 to 27 weeks of age. No significant difference in average water consumption was observed between three groups (CON, 4.4 ± 1.3; DM, 4.9 ± 2.9; DM + AKG, 4.5 ± 2.0 mL/day) at 26 weeks of age.

At 27 weeks old, body weights in both the DM and DM + AKG groups were significantly higher than that in the CON group (Table 1; *P* < 0.05). The weight (%body weight) of the soleus muscle in the DM group was significantly lower than that in the CON and DM + AKG group (Table 1; *P* < 0.05). The weights (% body weight) of the plantaris, TA, and gastrocnemius muscles in the DM and DM + AKG groups were lower than those in the CON group (Table 1; *P* < 0.05). The weights of the visceral, retroperitoneal, epididymal fats (% body weight) in both the DM and DM + AKG groups were significantly higher than those in the CON group (Table 1; *P* < 0.05). No difference in mesenteric fat weight (% body weight) was observed between three groups. The weight (% body weight) of BAT was significantly lower in the DM group than that in the CON group (Table 1; *P* < 0.05), while there was no significant difference in it between CON and DM + AKG groups (Table 1).

**Table 1.** Body weight and tissue weights relative to body weight

	CON (n = 7)	DM (n = 9)	DM + AKG (n = 10)
Body weight (g)	41.7 ± 5.2	51.9 ± 4.4^a^	50.4 ± 6.3^a^
Soleus muscles/BW (×100, %)	2.5 ± 0.2	2.3 ± 0.2^a^	2.5 ± 0.1^b^
Plantaris muscles/BW (×100, %)	6.3 ± 0.7	4.8 ± 0.6^a^	5.0 ± 0.5^a^
TA muscles/BW (×10, %)	1.6 ± 0.2	1.2 ± 0.1^a^	1.3 ± 0.1^a^
Gastrocnemius muscles/BW (×10, %)	4.1 ± 0.4	3.1 ± 0.3^a^	3.4 ± 0.4^a^
Visceral fat/BW (%)	2.3 ± 0.9	5.3 ± 1.5^a^	4.8 ± 1.2^a^
Retroperitoneal fat/BW (×10, %)	5.0 ± 2.9	11.5 ± 3.9^a^	10.0 ± 3.0^a^
Epididymal fat/BW (×10, %)	8.6 ± 3.2	25.8 ± 6.0^a^	22.4 ± 4.6^a^
Mesenteric fat /BW (×10, %)	1.0 ± 0.4	1.6 ± 0.6	1.6 ± 0.5
BAT/BW (×10, %)	2.8 ± 0.9	1.9 ± 0.5^a^	2.1 ± 0.6

Values are presented as the means ± SD (n = 7–10). ^a^ *P* < 0.05 compared with the CON group, ^b^ *P* < 0.05 compared with the DM group. CON, control; DM, diabetes; DM + AKG, diabetes with alpha-ketoglutarate supplementation.

### Blood substrate concentration

**Fig. 1.**
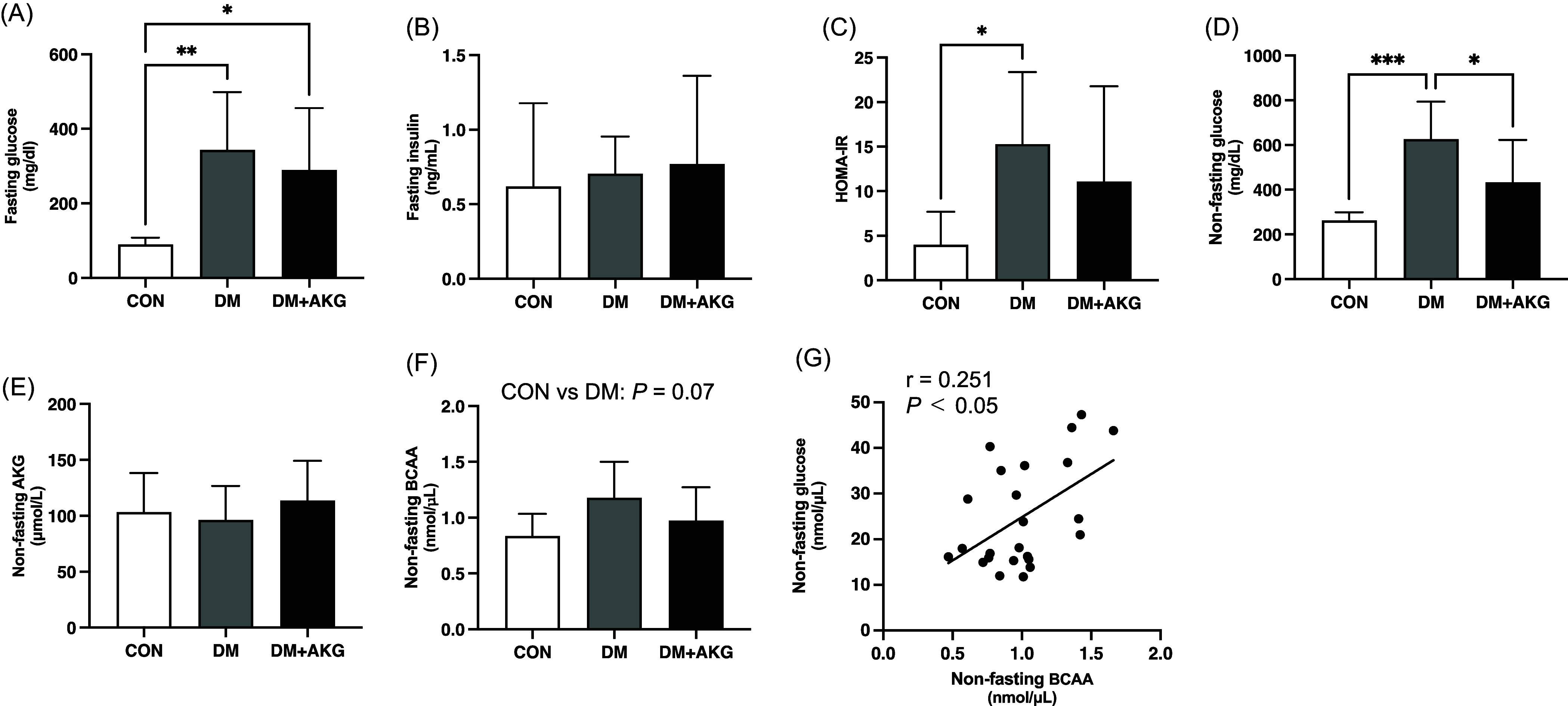
Fasting glucose (A) and insulin (B) levels in plasma, (C) homeostatic model assessment-insulin resistance (HOMA-IR), non-fasting glucose (D), AKG (E), BCAA (F), and concentrations in plasma and correlation between the non-fasting glucose and BCAA levels (G). Values are presented as the means ± SD (n = 7–10). *: *P* < 0.05, **: *P* < 0.01, and ***: *P* < 0.001 among groups. AKG, alpha-ketoglutarate; BCAA, branched-chain amino acid; CON, control; DM, diabetes; DM + AKG, diabetes with alpha-ketoglutarate supplementation.

The fasting glucose level was significantly higher in the DM and DM + AKG groups than that in the CON group (Fig. 1A, *P* < 0.05). There were no differences in fasting plasma insulin level among the three groups (Fig. 1B). The HOMA-IR was significantly higher in the DM group than that in the CON group (Fig. 1C, *P* < 0.05), while there was no significant difference between CON and DM + AKG groups (Fig. 1C). The non-fasting plasma glucose level was significantly higher in the DM group than that in the CON and DM + AKG groups (Fig. 1D, *P* < 0.05). There was no significant difference in the non-fasting AKG concentration between three groups (Fig. 1E). The non-fasting BCAA concentration in the DM group tended to be higher than that in the CON group (Fig. 1F, *P* = 0.07). Non-fasting plasma glucose level positively correlated with BCAA concentrations in all groups (Fig. 1G, *P* < 0.05, r = 0.251).

### GLUT4 levels in the muscles

**Fig. 2.**
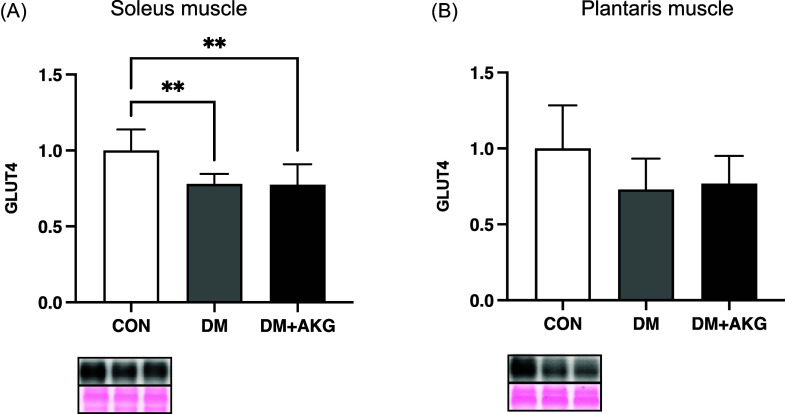
GLUT4 levels in the soleus (A) and plantaris (B) muscles. Values are presented as the means ± SD (n = 7–10). **: *P* < 0.01 among groups. GLUT4, glucose transporter 4; CON, control; DM, diabetes; DM + AKG, diabetes with alpha-ketoglutarate supplementation.

GLUT4 levels in the DM and DM + AKG groups were significantly lower than those in the CON group in the soleus muscle (Fig. 2A, *P* < 0.01). GLUT4 levels showed no significant differences between the groups in the plantaris muscle (Fig. 2B).

### GLUT4, PGC-1α, UCP1 and PRDM16 levels in the adipose tissues

**Fig. 3.**
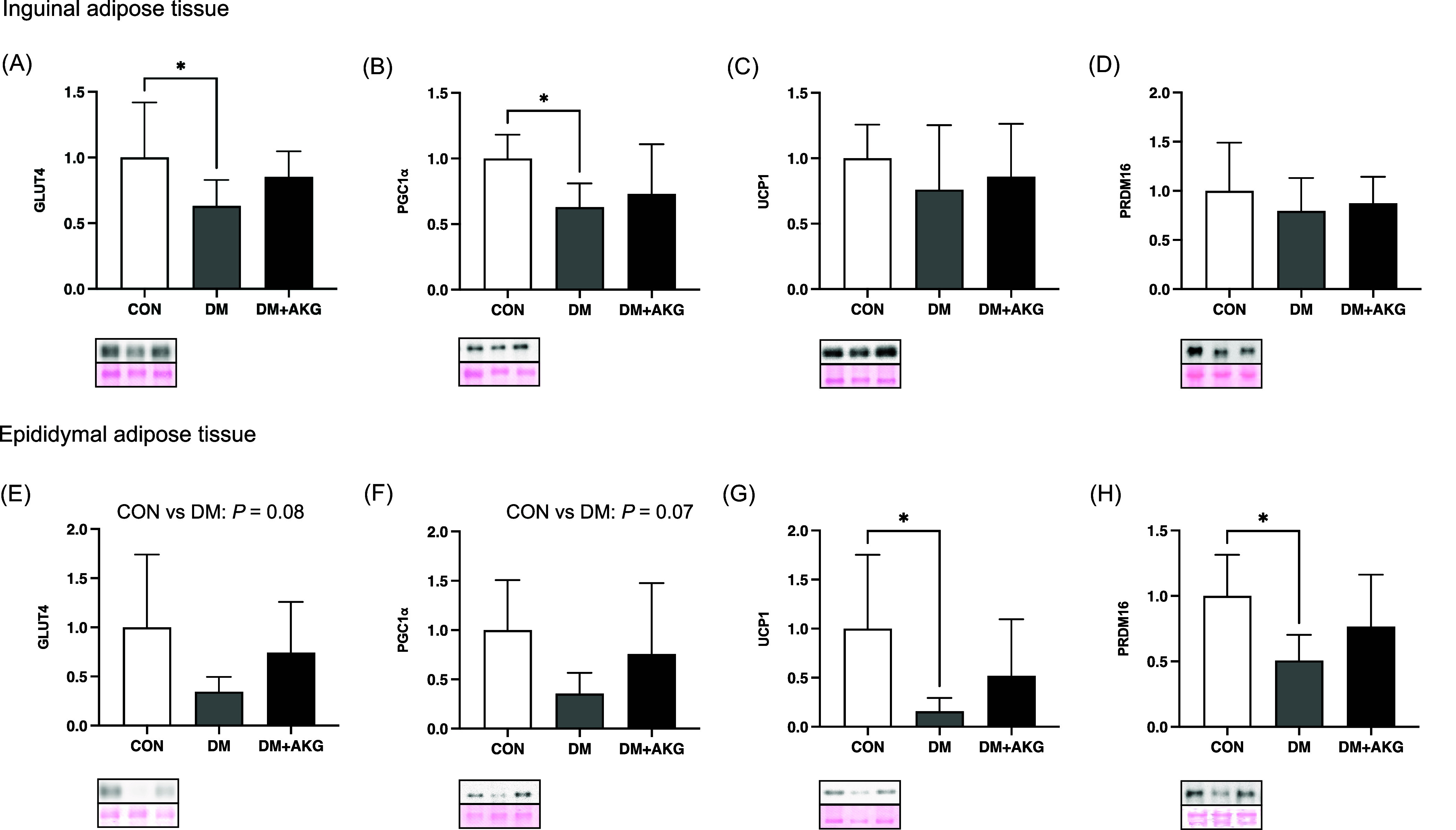
GLUT4, PGC-1α, UCP1 and PRDM16 levels in the inguinal (A)–(D) and epididymal (E)–(H) adipose tissues. Values are presented as the means ± SD (n = 7–10). *: *P* < 0.05 among groups. GLUT4, glucose transporter 4; PGC-1α, peroxisome proliferator-activated receptor γ coactivator 1 alpha; UCP1, uncoupling protein 1; PRDM16, PRD1-BF-1-RIZ1 homologous domain containing protein 16; CON, control; DM, diabetes; DM + AKG, diabetes with alpha-ketoglutarate supplementation.

In inguinal adipose tissue, GLUT4 and PGC-1α (a master regulator of mitochondrial biogenesis) levels in the DM group were significantly lower than those in the CON group (Fig. 3A and B, *P* < 0.05), while there was no significant difference in these levels between CON and DM + AKG groups (Fig. 3A and B). There were no differences in protein levels of UCP1 and PRDM16, a main regulatory factor in thermogenesis, among the three groups (Fig. 3C and D).

In the epididymal adipose tissue, GLUT4 and PGC-1α levels in the DM group tended to be lower than those in the CON group (Fig. 3E and F, *P* = 0.08 and 0.07, respectively). UCP1 and PRDM16 levels in the DM group was significantly lower than those in the CON group (Fig. 3G and H, *P* < 0.05).

### OXPHOS levels in the adipose tissues

**Fig. 4.**
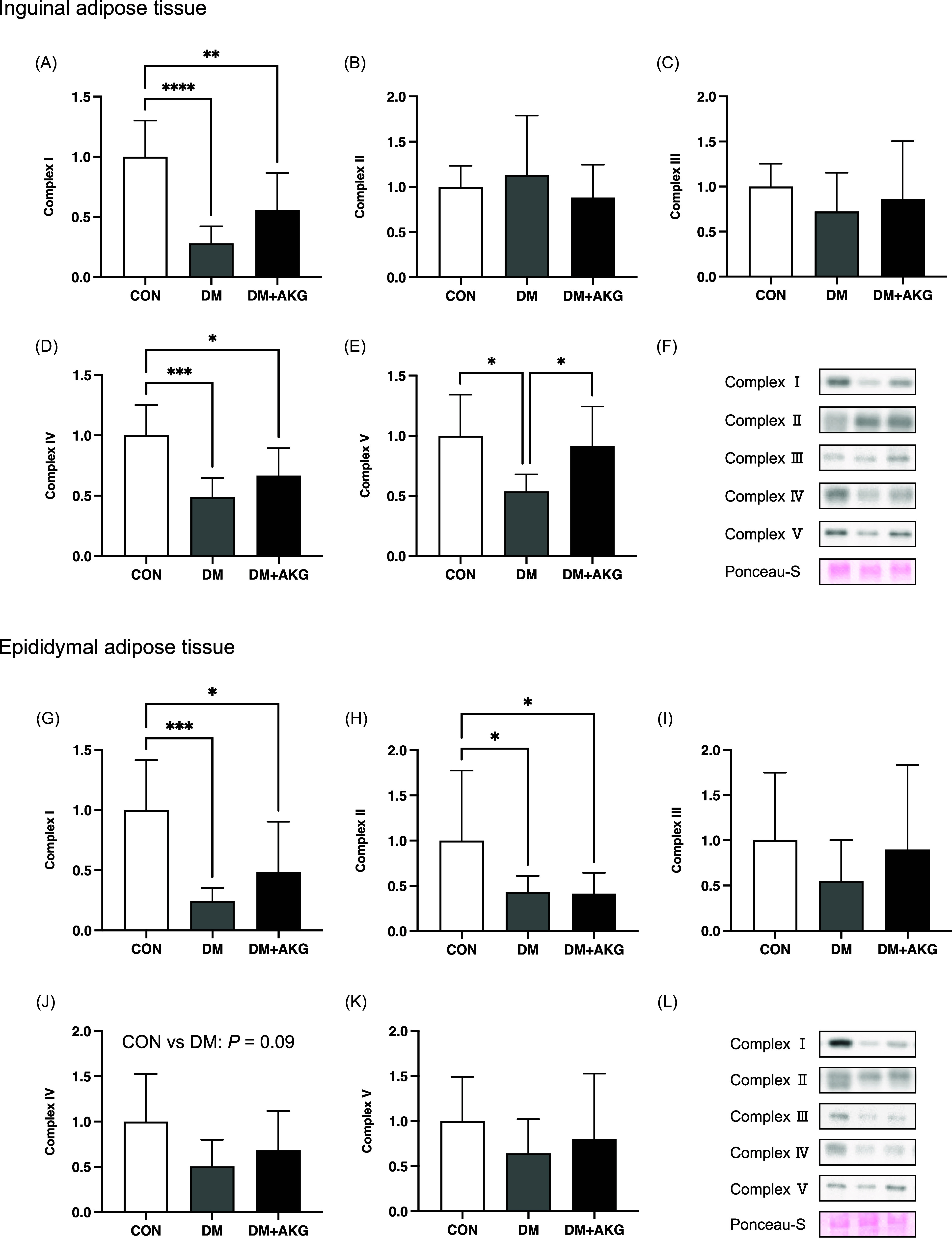
Mitochondrial proteins, complexes I-V, in the inguinal (A)–(F) and epididymal (G)–(L) adipose tissues. Values are presented as the means ± SD (n = 7–10). *: *P* < 0.05, **: *P* < 0.01, ***: *P* < 0.001 and ****: *P* < 0.0001 among groups. CON, control; DM, diabetes; DM + AKG, diabetes with alpha-ketoglutarate supplementation.

In inguinal adipose tissue, mitochondrial OXPHOS complex I levels in the DM and DM + AKG groups were significantly lower than that in the CON group in inguinal adipose tissue (Fig. 4A, *P* < 0.0001 and 0.01, respectively). There were no differences in complex II and III protein levels among the three groups (Fig. 4B and C). Complex IV levels in the DM and DM + AKG groups were significantly lower than that in the CON group in inguinal adipose tissues (Fig. 4D, *P* < 0.001 and 0.05, respectively). Mitochondrial complex V level in the DM group was significantly lower than those in the CON and DM + AKG groups in inguinal adipose tissues (Fig. 4E, *P* < 0.05).

In epididymal adipose tissue, mitochondrial complexes I and II levels in the DM and DM + AKG groups were significantly lower than those in the CON group (complex I, Fig. 4G, *P* < 0.001 and 0.05, respectively; complex II, Fig. 4H, *P* < 0.05). There was no difference in mitochondrial complex III level among the three groups (Fig. 4I). Mitochondrial complex IV level in the DM group tended to be lower than that in the CON group (Fig. 4J, *P* = 0.09). There were no differences in mitochondrial complex V level among the three groups (Fig. 4K).

### BCAT2 levels in the muscles and adipose tissues

**Fig. 5.**
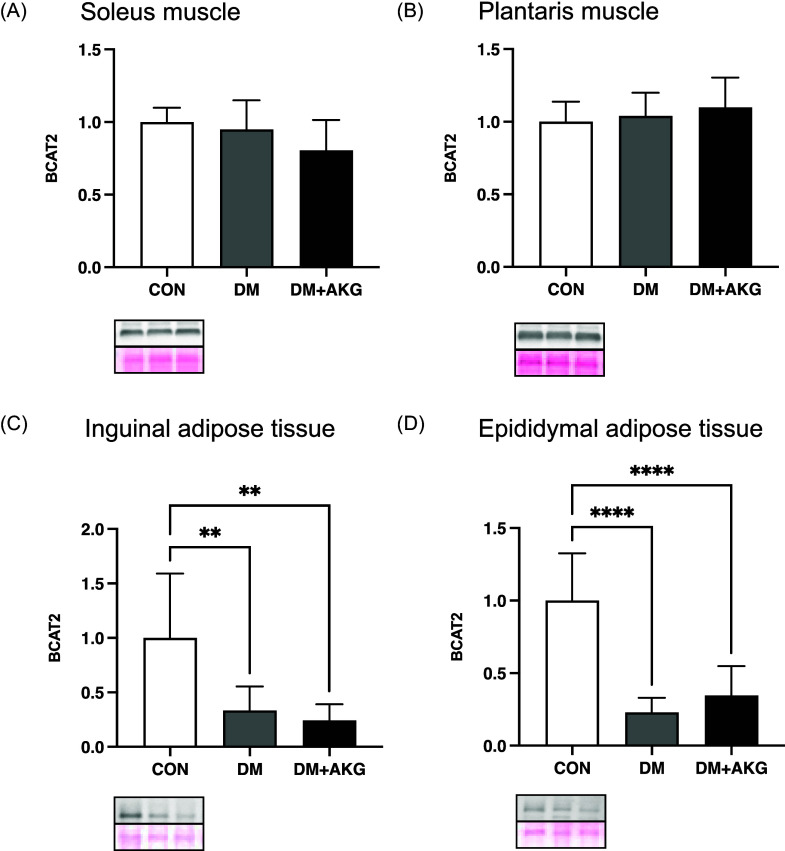
BCAT2 levels in the soleus (A) and plantaris (B) muscles and inguinal (C) and epididymal (D) adipose tissues. Values are presented as the means ± SD (n = 7–10). **: *P* < 0.01 and ****: *P* < 0.0001 among groups. BCAT2, branched chain amino acid transaminase 2; CON, control; DM, diabetes; DM + AKG, diabetes with alpha-ketoglutarate supplementation.

In the soleus and plantaris muscles, BCAT2 (an important enzyme in BCAA catabolism) levels showed no significant differences among all groups (Fig. 5A and B). In inguinal and epididymal adipose tissues, BCAT2 levels in the DM and DM + AKG groups were significantly lower than those in the CON group (Fig. 5C and D, *P* < 0.01).

## Discussion

We investigated the effects of AKG supplementation on glucose metabolism of STZ-HFD-induced diabetic mice. Supplementation with AKG improved the whole-body glucose metabolism and partially attenuated decreases in protein levels related to mitochondrial biogenesis and glucose transporters in adipocyte tissues in STZ-HFD-induced diabetic mice.

In this study, there was no difference in water intake between the three groups. Food intake increases in many animal models of DM, although food intake and water intake also decrease in HFD-induced DM models,^(34)^ such as this study. In general, an increase in food intake is correlated with an increase in water intake. Food intake and water intake decreased in the HFD-fed animals, probably due to the satiety of the HFD.

Previous study showed that the AKG supplementation enhances glucose metabolism;^(25,26)^ however, the adaptations of muscles and adipocyte tissues has not been well studied in diabetic mice. In this study, AKG administration did not increase the level of GLUT4 in soleus and plantaris muscles in DM. On the other hand, the present study showed that AKG supplementation attenuated decreases in PGC-1α and GLUT4 levels in inguinal and epididymal adipose tissues in STZ-HFD-diabetic mice. Previous study suggested that *Pgc-1α* mRNA levels significantly correlated with *Glut4* mRNA levels in adipose tissues.^(35)^ The upregulated PGC-1α due to AKG intake may induce the high GLUT4 levels in adipose tissue, and this adaptation resulted in attenuating the hyperglycemia of diabetic mice in this study. The estimated glucose uptake rate of the adipose tissue is approximately 7% of the dietary carbohydrate in healthy participants.^(36)^ However, the percentage of glucose uptake in the adipose tissue in obese participants was higher than that in non-obese participants (approximately 17% of the dietary carbohydrates) and it was similar to that in the muscle (approximately 19%) in obese participants. Previous study showed that adipose-specific deficiency of PGC-1α mice developed insulin resistance than control mice floxed PGC-1α allele when mice fed a HFD.^(37)^ These findings suggest that PGC-1α level in adipose tissue contributes to whole-body glucose metabolism via to GLUT4. In the present study, higher GLUT4 levels in adipose tissues after AKG supplementation might be involved in ameliorate HFD-STZ induced hyperglycemia. In addition to these adaptations, the DM-induced decrease in mitochondrial complex V protein levels in inguinal adipose tissue was significantly improved by AKG supplementation. Because complex V is essential for mitochondrial ATP production, its preservation suggests that AKG may also improve glucose homeostasis by maintaining mitochondrial energy metabolism.

As reported in a previous study,^(20)^ AKG supplementation attenuated decrease in UCP1 and PRDM16 levels in the epididymal adipose tissue. PRDM16, which strongly induces UCP1 expression through forming a transcriptional complex with PGC-1α,^(38)^ is highly expressed in adipose tissues and plays a critical role in their development and differentiation.^(39)^ Given these functions, PRDM16 has also been regarded as a promising target for DM treatment.^(39,40)^ However, such potential may not be directly applicable to classical type I DM, which is typically modeled by high-dose STZ treatment. In this context, UCP1 is a key protein expressed in adipose tissue that promotes thermogenesis by dissipating the mitochondrial proton gradient instead of generating ATP.^(41)^ Previous studies have shown that Ucp1-independent thermogenesis increases energy expenditure and attenuates obesity or DM.^(41,42)^ A previous study reported that activation of AMP-activated protein kinase (AMPK) improved brown fat thermogenesis impaired by obesity.^(43)^ This effect was associated with AMPK/PRDM16 role in promoting brown fat development by regulating gene expression through cellular levels of AKG.^(44)^ AKG supplementation also mitigated the decrease in BAT weight (% body weight) in DM. These results are consistent with the previous study showing that the dietary AKG promotes beige adipogenesis in middle-aged mice with HFD-induced obesity.^(20)^ Adipose tissues containing abundant mitochondria, similar as BAT, take up more glucose and fatty acids to increase the mitochondrial heat production.^(21)^ In this study, mitochondrial complex V in the inguinal adipose tissue and complex IV in the epididymal adipose tissue of the DM group decreased significantly or tended to decrease, respectively, compared with the CON group, accompanied by similar changes in PGC-1α (a master regulator of mitochondrial biogenesis) expression in these tissues. In contrast, no significant or tended differences were observed in the expression of mitochondrial complexes or PGC-1α between the CON and DM+AKG groups. These results suggested that AKG supplementation partially inhibited the decrease of mitochondrial complexes in adipose tissue in the diabetic mice. This adaptation of the adipose tissue is one of the characteristics of beige adipocytes. In this study, AKG supplementation partially rescued the decreased mitochondrial proteins levels in the adipose tissues in DM and it may result in ameliorating DM symptoms.

Previous studies suggested that elevated blood BCAA levels are related to insulin resistance.^(14–17)^ We hypothesized that AKG supplementation decreases high BCAA levels in DM, thereby improving hyperglycemia, as AKG plays an important role in BCAA catabolism in mitochondria. Considering that BCAA activate mechanistic target of rapamycin complex 1 (mTORC1),^(45)^ a reduction in BCAA levels may suppress its activation. Given that excessive mTORC1 activation has been implicated in the development of insulin resistance,^(46)^ it is possible that AKG supplementation alleviates insulin resistance by reducing elevated circulating BCAA levels in DM. Indeed, non-fasting circulating BCAA levels tended to increase in the DM group compared to those in the CON group although the difference was not significant, and the BCAA level correlated with the circulating glucose level. A recent review showed that treatment with leucine increased signaling markers of mitochondrial biogenesis in adipocytes and myocytes.^(47)^ This finding suggests that BCAA, particularly leucine, stimulates signaling associated with increased mitochondrial content. However, in this study, despite the tendency for elevated plasma BCAA levels in mice with DM, the mitochondrial complex protein in adipose tissue remained unchanged. It is also conceivable that AKG, by participating in BCAA transamination, could increase concentrations of branched-chain keto acids (BCKAs), some of which, such as α-ketoisocaproate, have been reported to promote insulin secretion in pancreatic β-cells.^(48)^ However, in this study, AKG intake did not show a significant effect on insulin secretion. This pathway could therefore represent a potential but as yet unconfirmed mechanism by which AKG might influence glucose homeostasis, and warrants further investigation.

Previous studies showed that the huge losses in BCAT2 protein, an enzyme in BCAA catabolism, were observed in the obesity rodents.^(49,50)^ These results suggest that there is possibility that decreased BCAA catabolism in adipose tissues contributes to the rise in plasma BCAA level in obesity and T2DM.^(15,49,50)^ Given that AKG has been reported to activate AMPK,^(51,52)^ which can regulate transcriptional coactivators such as PGC-1α,^(53)^ and that PGC-1α has been shown to upregulate BCAT2 expression in skeletal muscle,^(54)^ we hypothesized that AKG might influence BCAT2 expression indirectly through these upstream regulators. To investigate this possibility, we measured BCAT2 levels in both adipose tissues and muscles. There were no differences between three groups in the BCAT2 level in the muscle in this study. BCAT2 level was lower in the adipose tissues of the diabetic groups regardless AKG supplementation than those in the CON group. These results suggested that AKG supplementation did not rescue the BCAT2 level in adipose tissues.

In this study, AKG supplementation attenuated the decrease in soleus muscle weight caused by DM. This result agrees with a previous study that showed that AKG prevented muscle atrophy in a Duchenne muscular dystrophy mouse model^(55)^ and increased gastrocnemius and soleus muscle weights in mice.^(56)^ The mechanisms by which AKG rescues muscle atrophy in DM was unclear; thus, further studies are needed. The present study has additional limitations. First, we did not determine the concentration of glutamate formed from AKG by the enzyme glutamate dehydrogenase or produced through transamination of BCAA. Future studies should determine whether AKG affects plasma glutamate levels. Second, only plasma BCAA levels were measured, which may not sensitively reflect transamination activity. It is possible that more pronounced differences in BCAA levels could be observed in other organs, such as the liver, which plays a central role in amino acid metabolism. Future studies should determine whether AKG affects liver BCAA levels. Third, although HFD was used in combination with STZ, this model involves pancreatic β-cell damage. Previous studies have shown that AKG inhibits ATP synthase and activates AMPK.^(52,53,57)^ AKG improved systemic insulin sensitivity, possibly via AMPK activation,^(26,43)^ although in this study it showed no significant effect on insulin secretion. Therefore, it may be more appropriate to further evaluate the effects of AKG in models more specifically focused on insulin resistance. Nonetheless, it is also possible that AKG acts through other pathways, including potential effects on pancreatic function, to decrease non-fasting glucose level. Further studies are needed to explore these possibilities. Finally, although the AKG-induced changes in PGC-1α, GLUT4, UCP1, and PRDM16 levels in adipose tissues were limited to partial attenuation of DM-induced decreases, further work is needed to clarify their contribution to glucose metabolism.

In conclusion, non-fasting glucose was significantly improved by AKG supplementation in the present study. AKG supplementation partially attenuated the DM-induced decreases in PGC-1α and GLUT4 levels in adipose tissues, suggesting limited but meaningful effects on adipose tissue adaptations in diabetic mice. Taken together, these novel results indicate that AKG can improve whole-body glucose metabolism in STZ-HFD-induced diabetic mice.
